# Oxytocin and Addiction: Potential Glutamatergic Mechanisms

**DOI:** 10.3390/ijms22052405

**Published:** 2021-02-27

**Authors:** Megana Sundar, Devon Patel, Zachary Young, Kah-Chung Leong

**Affiliations:** Department of Neuroscience, Trinity University, San Antonio, TX 78212, USA; msundar@trinity.edu (M.S.); dpatel2@trinity.edu (D.P.); zyoung@trinity.edu (Z.Y.)

**Keywords:** oxytocin, glutamate, addiction, reward processing, dopamine

## Abstract

Recently, oxytocin (OXT) has been investigated for its potential therapeutic role in addiction. OXT has been found to diminish various drug-seeking and drug-induced behaviors. Although its behavioral effects are well-established, there is not much consensus on how this neuropeptide exerts its effects. Previous research has given thought to how dopamine (DA) may be involved in oxytocinergic mechanisms, but there has not been as strong of a focus on the role that glutamate (Glu) has. The glutamatergic system is critical for the processing of rewards and the disruption of glutamatergic projections produces the behaviors seen in drug addicts. We introduce the idea that OXT has direct effects on Glu transmission within the reward processing pathway. Thus, OXT may reduce addictive behaviors by restoring abnormal drug-induced changes in the glutamatergic system and in its interactions with other neurotransmitters. This review offers insight into the mechanisms through which a potentially viable therapeutic target, OXT, could be used to reduce addiction-related behaviors.

## 1. Introduction

The neuropeptide oxytocin (OXT) has been found to have effects on addictive behavior, in addition to having prosocial and anxiolytic effects [1,2,3]. The most notable impact that OXT has on addiction is reducing drug-seeking behavior, as measured by experimental paradigms, such as self-administration and conditioned place preference [4]. The reward circuit seems to be an integral part of the mechanisms by which OXT attenuates drug-seeking behavior, although the specific mechanisms are still being investigated [5,6,7]. It is likely that OXT alters reward processing to take away the desire or motivation for a reward, or to disassociate the rewarding properties of the reward itself. Within the reward circuit, glutamate (Glu) and dopamine (DA) projections are primarily responsible for reward processing [2,7,8,9]. Understanding how OXT affects glutamatergic and dopaminergic pathways is crucial in order to advocate for the development of this compound into a treatment for addiction. This review focuses on the molecular mechanisms through which OXT influences Glu transmission, as the role of Glu in addiction is one that has not received much attention, despite its significance. 

### 1.1. The Endogenous Oxytocin System

OXT is a neurohormone consisting of nine peptides and is synthesized in the paraventricular nucleus (PVN) and supraoptic nucleus (SON) of the hypothalamus. The PVN and SON have magnocellular and parvocellular neurons, which release OXT. Parvocellular neurons in the PVN and SON are responsible for the central diffusion of OXT that helps to regulate behavioral responses and reward processing [10,11]. However, most of the OXT releasing neurons in the PVN and SON are magnocellular, which project to the posterior pituitary gland, allowing OXT to be released peripherally [11,12]. Peripherally acting OXT contributes to bodily functions mainly for the reproductive system, stress response, and pair bonding [10]. In the reproductive system, OXT is involved in processes such as giving birth or producing milk during lactation while, for social or emotional stress, it relieves anxiety by facilitating social interactions [13]. The release of OXT into the peripheral system can be triggered by events such as labor, nipple stimulation, and psychosocial stress [1]. Changes in endogenous OXT levels are also observed following the consumption of certain drugs of abuse. For instance, methamphetamine administration increases plasma OXT levels in juvenile rats [14].

The oxytocin receptor (OXTR) is a G-protein coupled receptor (GPCR) that is found throughout the body and the brain. The OXTR can couple to the G_q/11_ or G_i_ protein to initiate the phospholipase C signaling pathway that enhances neuronal firing [12]. This receptor is also able to bind to vasopressin, another neuropeptide, with similar affinity [11]. Peripherally, OXTRs are found in tissues of the kidneys, pancreas, heart, uterus, and mammary glands [1,11]. Centrally, OXTRs are found in brain regions that regulate reward processing, mood, and social behavior. The localization of OXTRs is similar across rat and human brains, but there are slight notable differences between these species. Both rats and humans have OXTRs in the central amygdala (CeA), substantia nigra (SN), paraventricular thalamic nucleus, olfactory nucleus, lateral mammillary nucleus, and ventral pallidum [12,15]. However, only humans seem to have OXTRs in the globus pallidus, basal nucleus of Meynert, medial preoptic area, and dorsal raphe nucleus. Rats have OXTRs in the ventral tegmental area (VTA), nucleus accumbens (NAc), prefrontal cortex (PFC), bed nucleus of the stria terminalis, PVN, SON, ventromedial hypothalamus, and hippocampus [12,16,17]. It should also be noted that there are sex-based differences in the expression of OXT and OXTRs, with female rats exhibiting higher OXT concentrations and male rats showing higher OXTR expression [18].

### 1.2. The Reward Circuit

The reward circuit, or the mesocorticolimbic system, is responsible for regulating the association between substances and reward. Rewarding substances can be drugs of abuse (cocaine, methamphetamine, opiates, etc.) or natural rewards, such as food, sex, and social interactions [19]. Glu, DA, and γ-aminobutyric acid (GABA) transmission between the structures that comprise the reward circuit is how the brain recognizes rewards and stimulates motivation to obtain them [20].

The VTA is one of the key structures in the reward circuit. It consists mostly of DA neurons and receives glutamatergic inputs from the medial prefrontal cortex (mPFC), lateral habenula (LHb), lateral hypothalamus (LH), and laterodorsal tegmentum [21]. VTA cells also receive GABAergic signals from the NAc and ventral pallidum. Within the VTA, there are GABAergic interneurons which project to VTA DA neurons to regulate DA release [21]. A population of Glu neurons also resides in the VTA, as discovered by expression of the vesicular glutamate transporter 2 (VGLUT2) [22]. 

The VTA is pivotal because it is the start of the two major pathways of the reward circuit: the mesolimbic and mesocortical pathways. In the mesolimbic pathway, VTA DA neurons project primarily to the NAc, and they also reach the amygdala and hippocampus. The NAc is another key structure that is known for having a large role in reward processing because of its localized DA buildup. The NAc mostly contains medium spiny neurons and is abundant with D1- and D2-like DA receptors [20]. Aside from receiving DA signals from the VTA, the NAc receives glutamatergic inputs from the amygdala, PFC, ventral hippocampus, and mediodorsal thalamus [21]. In the mesocortical pathway, the VTA sends dopaminergic outputs to cortical brain regions, such as the mPFC, basal ganglia, and anterior cingulate cortex (ACC). Although the mesolimbic system is often considered as the main reward pathway, the mesocortical pathway involves structures with important roles in reward processing. Together, these pathways constitute the reward circuit, integrating aspects of reward processing like motivation, movement, memory, and cognition [23].

In addition to the mesocorticolimbic system, the fronto-striatal neural circuit is associated with reward processing. In this circuit, the ventral striatum sends GABAergic projections to the basal ganglia, which then projects to cortical regions (most importantly, the orbitofrontal cortex and mPFC) via the thalamus. The ventral striatum also receives dopaminergic and glutamatergic inputs from the basal ganglia and cortical regions, respectively [24]. This network specifically plays a role in reward responsivity, goal-directed behavior, and incentive-based learning [25]. Many factors can modulate the fronto-striatal circuit’s activity to reward or reward-predictive cues, such as the effort required to obtain a reward, the magnitude of the reward, and delay in time until the reward is gained [26].

**Figure 1:**
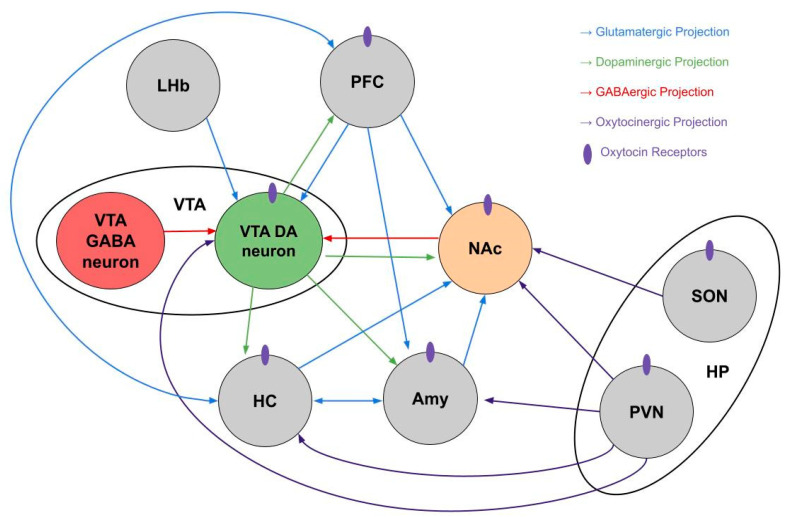
Diagram of the key components and pathways of the classic reward circuit, its commonly associated structures, and its interactions with the endogenous oxytocin system. Rodent oxytocin receptors are found throughout this circuit and influence glutamatergic and dopaminergic systems. VTA = ventral tegmental area; NAc = nucleus accumbens; Amy = amygdala; PFC = prefrontal cortex; PVN = paraventricular nuclei; SON = supraoptic nuclei; HP = hypothalamus; LHb = lateral habenula; HC = hippocampus; GABA = γ-aminobutyric acid; DA = dopamine.

The endogenous OXT system interacts with the reward circuit in several locations (Figure 1). Oxytocinergic projections from parvocellular neurons of the PVN travel to midbrain DA neurons, specifically in the VTA, NAc, and SN [27,28]. All of these structures contain a high density of OXTRs to which the neuropeptide binds. Peris et al. (2017) found that approximately 50% of the VTA neurons that express OXTRs are glutamatergic [29]. Another report determined that around 46% of OXTR-expressing neurons in the PFC are glutamatergic [30]. Endogenous OXT projections to the reward circuit are thought to mediate the rewarding effects of social bonding and affiliative behavior [12]. The presence of OXTRs in central components of the mesocorticolimbic system is important for another function of OXT, one that is the focus of this review: its ability to reduce addictive behaviors.

### 1.3. Reward Processing and Addiction

The reward circuit is heavily implicated in the development and maintenance of an addiction. Addiction is caused by a substance or behavior that provides pleasure and/or reduces pain, even though it may result in harmful physical and mental consequences, and is unable to be controlled [31]. Typical addictive substances are drugs of abuse (cocaine, alcohol, methamphetamine, etc.) and, to a lesser extent, natural rewards such as food; behavioral addictions include sexual behavior and gambling [32]. For the purposes of this review, substance addiction will be the center of interest. 

Addictive substances are highly rewarding, so they act as incentives, such that when they are no longer present in the brain, reward-seeking behavior is stimulated to acquire the pleasure and fulfillment provided by these substances again [33]. Feeding an addiction thus leads to a dangerous cycle of consuming substances that are rewarding but harmful, and then having cravings which incite reward-seeking behavior. The reward processing system is activated to perceive the rewarding properties of a substance and provoke reward-seeking behavior [21]. Another function of reward processing is pairing a reward with a stimulus through associative learning. This association causes a stimulus to act as a reward-predictive cue, which provokes motivation and cravings by having incentivizing properties [34,35]. Reward-predictive cues in animal-based experiments are typically an object or context, such as a lever or chamber which has been paired with the reward through conditioning. Aversive stimuli, such as physical or emotional stress, can also be used to promote reward-seeking behavior [36]. In humans, reward-associated stimuli, whether positive or aversive, are more subjective and personal, such as a memory, smell, location, or stress-inducing event [37]. These stimuli serve as triggers for people with drug addiction and can be the cause for relapse after a long period of abstinence. 

Mesocorticolimbic system structures have specific roles in reward processing, and therefore, addiction. Since the VTA is the origin of the reward circuit pathways, it has implications in many aspects of addiction. Activation of the VTA can be traced to specific events, such as the intake of an unexpected reward, seeing a reward-predictive cue, and exposure to natural rewards [38,39]. Once the VTA is stimulated, it sends signals to encode the properties of rewards and their associated stimuli [40]. Behavioral responses to a reward are also a result of the activated VTA, projecting to other structures in the reward circuit. Exogenously delivered opioids, for example, bind to opioid receptors on the VTA, which promotes a large release of DA to the NAc, resulting in a rewarding feeling and behaviors such as locomotor hyperactivity and drug-seeking [41,42]. 

The NAc is another structure that is heavily involved in addiction, which is expected due to its major role in the reward circuit. The NAc is composed of two components: the core and the shell. These subregions differ in their functions, effects on behavior, and cellular morphology. Functions of the NAc core include evaluating a substance as rewarding or aversive and motivating drug-seeking behaviors, such as initiating movement towards a reward. The shell, on the other hand, is associated with reward prediction and incentive learning [43,44,45,46]. The NAc shell is also associated with the habituation of the DA response to natural rewards [47]. However, lesions of DA terminals in the mPFC abolish this habituation of natural rewards in the shell, suggesting that this process requires intact DA transmission from the mPFC [48]. Interestingly, drugs of abuse do not produce adaptive responses of DA release to the NAc shell, similar to habituation or inhibition by predictive stimuli [49]. The NAc core and shell also differs in the projections that they receive, such as glutamatergic projections from the mPFC, which are associated with mediating the seeking and planning of action to obtain rewards and vary between these regions. Infralimbic mPFC, cells project to the NAc shell, while the prelimbic mPFC sends projections to the core [21]. Lesions to the NAc shell attenuate the psychostimulant effects of cocaine, but do not disrupt cocaine-seeking behavior, validating that each subregion has different roles when processing drugs of abuse [50]. 

Drugs of abuse interact with the reward circuit in various ways, causing severe maladaptations to its reward processing function. This dysfunction induces abnormally strong cravings and drug-seeking behaviors, thus initiating the cycle of addiction. Most drugs cause maladaptations to the mesolimbic system by substantially increasing extracellular DA, which encodes the substance with a highly rewarding value, and/or they influence changes in Glu and GABA transmission to prompt drug-seeking. For example, cocaine generates its stimulating effects by inhibiting the reuptake of DA by dopamine transporters, leading to abnormally high synaptic DA levels and a greater stimulation of DA receptors in the NAc [51,52]. This encourages persistent drug-seeking behaviors in animals, such as lever presses for cocaine. Methamphetamine impairs the reward circuit by inhibiting monoamine metabolism inside the cell and triggering the release of additional DA from presynaptic neurons [53,54]. Ethanol may have indirect effects on DA signaling and reward processing by blocking Glu transmission in the hippocampus, CeA, and frontal cortex, while promoting GABA firing in the basolateral amygdala [55], while also indirectly interacting with DA signaling from VTA neurons by influencing opioid and GABA signaling in upstream projections [56,57]. Recent evidence has suggested that natural rewards, such as food, sexual behavior, and social interactions, can also affect the reward circuit to elicit cravings, the impairment of control, and substance-seeking [32]. Natural rewards are believed to affect reward processing in a similar way to drugs of abuse, seeing as they activate the same reward circuit structures implicated in drug addiction [23]. In addition to Glu, DA, and GABA, other neurotransmitters that have an important role in drug dependence are serotonin (5-HT) and endocannabinoids. The 5-HT projections and its interactions with DA are especially important in cocaine addiction [58]. Microdialysis studies of endocannabinoids have demonstrated that they are released via dopaminergic signaling and are involved in regulating synaptic plasticity related to addiction development [58]. It is essential to recognize the roles that these neurotransmitters and their interdependence have in addiction, even though the focus of this review will be on Glu, Da, and GABA. 

### 1.4. Drugs of Abuse and Oxytocin

OXT counteracts the effects of rewarding substances, as seen by its ability to reduce drug-induced behaviors, drug-seeking behaviors, and cravings for addictive substances. OXT attenuates behaviors induced by cocaine, such as locomotor hyperactivity, repetitive behaviors (e.g., sniffing), and behavioral sensitization to cocaine in rodents [59]. OXT (0.1, 0.3, 1, 3 mg/kg; i.p.) also dose-dependently reduced lever presses for cocaine in male rats in a self-administration paradigm [60]. Reinstatement behavior, similar to drug relapse, is influenced by OXT administration as well. The administration of OXT into the NAc decreased the cued reinstatement of cocaine self-administration [30]. 

Similar effects of OXT are found for other drugs of abuse. Methamphetamine seeking, as measured by active lever presses, was decreased dose-dependently by systemic OXT treatment [61]. Peripheral (0.6 mg), intracerebroventricular (0.1, 0.5, and 2.5 μg), and NAc core and subthalamic nucleus (0.6 mg) injections of OXT all reduced the acquisition of methamphetamine-conditioned place preference (CPP) [8,62]. Additionally, OXT attenuated both drug-primed reinstatement [61,63] and the cue-induced reinstatement of methamphetamine [64]. When directly injected into the NAc core, OXT (1.5 and 4.5 pmol) dose-dependently reduced meth-primed reinstatement [65]. Regarding opioids, both peripheral and central treatments of OXT have successfully attenuated opioid tolerance in rodents, specifically to analgesic morphine and heroin [66]. In heroin-tolerant rats, a single dose of OXT (0.05, 0.5, and 5 µg; s.c.) was all that was required to reduce heroin self-administration and block the expression of heroin tolerance. However, this effect was not found in heroin-naive rats [67,68]. The acute intracerebroventricular administration of OXT (1 µg/5 µL) reduced alcohol self-administration and prevented the ethanol-induced release of DA in the NAc in rats both chronically exposed and naive to ethanol [69]. 

Looking at the effects of OXT on behaviors induced by natural rewards, peripheral (up to 6 mg/kg; i.p.) and intracerebroventricular (1–10 µg) OXT administration dose-dependently reduces food intake in food deprived rats [70]. Another study by Mullis and colleagues (2013) found that OXT directly injected into the VTA decreased the consumption of a sucrose solution. They also discovered that OXTR antagonists inhibited this effect and returned sucrose intake to normal levels, establishing that OXT attenuates sucrose intake via OXTRs in the VTA [71]. Additionally, the systemic administration of OXT was found to attenuate the expression, but not the acquisition, of sucrose CPP [72]. These findings, which demonstrate that OXT can decrease the seeking and intake of natural rewards, suggest that the neuropeptide could be used to reduce the overconsumption of food and sugar caused by maladaptive processes in reward processing. Thus, OXT may have a role in combating diseases associated with the overconsumption of food and sugar, such as diabetes and cardiovascular disease.

Several clinical trials have been initiated to test whether OXT curbs drug-seeking in humans. Pedersen and colleagues (2013) had alcohol-dependent subjects undergo alcohol detoxification with the treatment of lorazepam, as needed, and were to either receive intranasal OXT (24 IU; twice daily for three days) or a placebo. The subjects who received OXT demonstrated fewer withdrawal symptoms and used less lorazepam than the control group [73]. Another clinical trial found that a single intranasal OXT administration (40 IU) reduced drug cravings, stress, and anxiety in cannabis-dependent individuals [74]. 

Clearly, OXT has a profound effect on addictive behaviors and is a promising candidate to be a therapeutic for addiction. However, the mechanisms by which OXT reduces drug-induced behaviors are not well-established. This review aims to examine how OXT impacts maladaptive changes in the reward circuit, specifically focusing on its interactions with Glu and glutamatergic pathways to attenuate reward-seeking behaviors.

## 2. Glutamate and Addiction

Although DA is a prominent focus in studies of addiction, research into Glu’s role in addiction is growing. It appears that DA has a primary role in the beginning of the addictive cycle, while Glu is a greater factor in the later parts of the cycle (reinstatement and relapse). Glu may also be involved in mediating the effects of natural rewards [75]. This section will discuss the endogenous glutamatergic system, the role of Glu in addiction, interactions between DA and Glu in the reward circuit, and how drugs of abuse impact Glu transmission. Examining the functions that Glu has in reward processing is crucial to understanding the mechanisms involving the glutamatergic system through which OXT attenuates drug-seeking behaviors. 

### 2.1. Overview of the Glutamatergic System

Glu is the primary excitatory neurotransmitter in mammalian brains and is involved in a variety of processes, including learning, memory, and reward processing. Drugs of abuse alter Glu transmission and activity by influencing the amount of Glu transmission and can do so by interacting with the various Glu receptors. There are two main categories of Glu receptors: ionotropic and metabotropic. Ionotropic receptors (α-amino-3-hydroxy-5-methyl-4-isoxazolepropionic acid [AMPA], N-methyl-D-aspartate [NMDA], and kainate) are ligand-gated ion channels that alter cation (Ca^2+^, Na^+^) flow into and out of the cell; metabotropic receptors are GPCRs that activate or inhibit second messenger signaling cascades [76]. 

There are eight different subtypes and three different groups of metabotropic Glu receptors (mGluRs) that are separated by their signal transduction pathways and homology sequence. Typically, group 1 receptors (mGluR1 and mGluR5) are stimulatory, whereas Group 2 (mGluR2 and mGluR3) and Group 3 (mGluR4, mGluR6, mGluR7, and mGluR8) are inhibitory [77,78]. Group 1 receptors are predominantly postsynaptic receptors, Group 2 are both pre- and postsynaptic, and Group 3 are presynaptic autoreceptors [79,80,81]. These different groups of receptors are implicated in different parts of the addictive cycle. Group 1 receptors are important in drug reinforcement and Group 2 receptors are involved in neuroplasticity induced by chronic drug use or aversion during withdrawal; it is unclear how Group 3 receptors are involved in similar behaviors [80]. For the purposes of this paper, we will discuss OXT’s effect on Group 1 and Group 2 receptors.

### 2.2. Glutamate’s Role in Addiction

Glu transmission in the reward circuit, specifically in the NAc, has a role in the decision-making process of obtaining rewards by invoking motivational and emotional responses associated with the stimuli, determining the attention level allotted to stimuli, inhibiting impulsive behavior, and providing contextual information [75,82]. The NAc receives glutamatergic projections mainly from the PFC and amygdala, but also from the hippocampus and thalamic nuclei [75,83]. The glutamatergic projections from the PFC to the NAc are highly implicated in drug-seeking behaviors, especially relapse and reinstatement [84]. In fact, it is these projections that initiate cocaine-induced reinstatement [85]. Specifically, the activation of projections from the prelimbic PFC to NAc is essential for reinstatement behavior following cocaine extinction, while the inactivation of infralimbic PFC to NAc projections has the same effect [21].

Context-specific aspects of reward seeking appear to be more dependent on glutamatergic transmission [86]. Studies indicate that ionotropic Glu receptors are especially involved in drug-seeking behaviors mediated by drug-seeking cues. For instance, the microinfusion of an AMPA/kainate receptor antagonist into the NAc core, but not the shell, dose-dependently reduced lever presses for cocaine [87]. It is noteworthy that an NMDA receptor antagonist decreased cocaine-seeking behavior when infused into the NAc shell [87]. There is strong evidence that that Glu transmission in the NAc is a primary determinant of relapse [88]. However, the NAc core and shell subregions have different roles in the mediation of relapse. The shell seems to be responsible for context-induced relapse, whereas the core is essential for cue-induced and drug-primed reinstatement [85,89,90]. Additionally, the infusion of an AMPA antagonist, and not an NMDA antagonist, into the medial NAc blocks reinstatement for cocaine [91]. 

The Glu homeostasis theory offers another idea as to how Glu is implicated in addictive behaviors. This theory states that an imbalance between synaptic and extracellular Glu alters neuroplasticity in the corticostriatal pathway, thus impairing the ability to control drug seeking [92]. Excitatory amino acid transporters (EAATs), VGLUTs, and cystine-glutamate exchangers are components that are critical for maintaining homeostasis. EAATs and VGLUTs clear Glu from the synapse, while the cystine-glutamate exchangers transport it back into the extrasynaptic space [75]. Repeated exposure to drugs of abuse causes changes in the function of these components, therefore, disrupting the balance of synaptic Glu and promoting addictive behaviors [93]. The Glu homeostasis hypothesis is supported by a study from Baker et al. (2003), which displayed that restoration of extracellular Glu and stimulation of cystine and Glu exchange prevented cocaine-primed reinstatement [94]. 

### 2.3. Glutamate and Dopamine Interactions

The dopaminergic projections in the brain, especially in the reward circuit, are recognized as having a significant role in reward processing and addiction. DA released in the mesocorticolimbic system is utilized to encode the value of a reward, create incentive salience, facilitate reward-stimulus pairings, and anticipate a reward [95]. The release and utilization of DA are regulated by D1-like or D2-like GPCRs to appropriately process rewards. The D1-like receptors bind to stimulatory G-proteins and activate adenylyl cyclase, while D2-like receptors act oppositely by binding to inhibitory G-proteins [96]. D2-like receptors are commonly found as inhibitory autoreceptors that regulate dopaminergic activity [97]. 

DA transmission can be regulated by glutamatergic afferents and, conversely, DA can influence Glu transmission via inputs to glutamatergic neurons. This interdependence of Glu and DA transmission is critical for regulating various aspects of reward processing and addictive behaviors. Many glutamatergic projections originating in limbic, cortical, and subcortical structures innervate DA neurons in the VTA and the NAc. Glutamatergic projections to the VTA come primarily from the PFC, but some stem from the amygdala, hippocampus, LHb, LH, or ventral pallidum [98,99,100]. These glutamatergic inputs to the VTA help to modulate the phasic firing of the dopaminergic neurons directly or indirectly [101]. For instance, glutamatergic projections from the LHb to GABAergic neurons in the VTA inhibit the firing of VTA DA neurons [102]. Additionally, one study found that excitation of glutamatergic afferents from the hippocampus to NAc subsequently resulted in increased firing of VTA DA neurons [103]. Both metabotropic and ionotropic receptors have a large role in regulating DA levels. Administration of mGluR2 and mGluR3 agonists reduced extracellular DA dose-dependently, while an antagonist of mGluR2/3 increased DA [104]. Meanwhile, agonists of excitatory metabotropic receptors enhanced DA release in the NAc [105]. Ionotropic receptor antagonists decreased extracellular striatal DA in vivo, and agonists promoted DA release in vitro [106,107]. These studies suggest that Glu receptors are involved in regulating tonic concentrations of DA. 

The modulation of Glu transmission by dopaminergic projections is often exhibited by the effects of psychostimulants. Cocaine and methamphetamine block the reuptake of DA by binding to the DA transporter. This increases synaptic DA levels, which activates D1 receptors and enhances Glu transmission [108]. In addition, D1 receptor stimulation increases the surface expression of the GluR1 (or GluA1) subunit of the AMPA receptor on NAc neurons; this subunit is essential for LTP [109]. Another study found that D1 receptors and NMDA receptors interact with each other to retain a sufficient concentration of D1 receptors in the Glu synapses of the hippocampus [110]. 

### 2.4. Drugs of Abuse and Glutamate

Drugs of abuse greatly increase Glu and DA levels in the synapse and alter the transmission of these neurotransmitters using various mechanisms. As described previously, psychostimulants increase Glu transmission indirectly through interactions with DA [75]. Some studies show that alcohol can inhibit presynaptic Glu release and postsynaptic NMDA-mediated Glu transmission. However, other studies found Glu levels to increase after alcohol administration, possibly due to the activation of D1 receptors or inhibition of GABAergic interneurons that project to presynaptic Glu neurons [75]. Meanwhile, heroin activates mu-opioid receptors to enhance postsynaptic NMDA-mediated Glu transmission [111]. Heroin can also increase Glu signaling via dopaminergic interactions and the inhibition of GABA neurons, similarly to alcohol [75]. Methamphetamine self-administration impacted Glu transmission by decreasing the AMPA/NMDA ratio in the mPFC, which was driven by an increase in NMDA receptor currents and in the surface expression of the GluN2B subunit [112].

After withdrawal from alcohol, cocaine, nicotine, and heroin, NAc core astrocytes exhibit a decrease in glutamate transporter 1 (GLT-1) levels [113]. Additionally, there is an increase in postsynaptic AMPA receptor function, a downregulation of inhibitory Group 2 mGluRs, and/or upregulation of the activator of G-protein signaling 3 (AGS3) after withdrawal from alcohol, cocaine, heroin, or methamphetamine [113,114]. Cocaine alters AMPA receptor function in the NAc by upregulating its surface and synaptic receptor levels; it also decreases the surface expression of mGluR2 [113]. However, it should be noted that the effects that drugs have on corticostriatal Glu neuroplasticity can be dependent on various facets of the experimental paradigms used, such as contingency and duration [114,115].

Drug-induced alterations in Glu transmission play an important role in the behavioral aspects of addiction. Stimulant drugs have been shown to induce behavioral sensitization via glutamatergic activity, especially by acting on ionotropic (mostly NMDA) and metabotropic receptors in the VTA and NAc [116,117,118]. Compulsive drug-seeking behavior and the maladaptive formation of drug-associated memories results from NMDA receptor plasticity in the NAc and excessive Glu transmission from the PFC to the NAc [119,120]. Therefore, Glu is crucial in the neurophysiological and behavioral consequences of drugs of abuse.

## 3. Oxytocin and Glutamate

Administration of OXT can influence Glu levels in the reward circuit, suggesting that the neuropeptide may attenuate addictive behaviors by acting on the glutamatergic system. This is supported by findings which show that Glu and GABA cells are predominantly located in the medial fascicular and rostrolinear nuclei of the VTA, whereas DA cells are found in the lateral regions of the VTA, and OXT administration results in increased VTA firing rates with around 76% of the medial neurons responsive to OXT, compared to 28% in lateral areas [121,122]. Thus, the medial projections from the VTA to NAc, which are likely to be glutamatergic are also likely to be involved in oxytocinergic mechanisms. Seeing as OXTRs are found on dopaminergic, glutamatergic, and GABAergic cells in the VTA [29], and these receptors have been localized in regions implicated in drug-induced maladaptive Glu transmission, similar to the NAc (core and shell) and PFC [12,17], there is further support that OXT’s mechanisms involves a glutamatergic pathway.

A few studies have shown further evidence of Glu transmission being influenced by OXT treatment (Table 1). For instance, Qi et al. (2012) demonstrated that OXT counteracted methamphetamine-induced increase in Glu in the mPFC and decrease in Glu in the dorsal hippocampus [123]. In addition, OXT decreased extracellular Glu levels in the mPFC caused by stress-induced reinstatement of methamphetamine CPP [8]. Notably, both of these effects were reversed in the presence of an OXTR inhibitor. Here, we propose several mechanisms explaining how OXT affects glutamate transmission to attenuate drug seeking, all of which involve restoring drug-induced maladaptations to the reward circuit.

### 3.1. Ionotropic and Metabotropic Glutamate Receptors

It is possible that OXT attenuates abnormal Glu transmission by modifying the activity of Glu receptors. In examining its interactions with ionotropic receptors, OXTRs were found to reduce methamphetamine-induced increases in the NMDA NR1 subunit in the PFC [123]. OXT administration also opposed cocaine-induced decreases in the phosphorylation of the GluR1 AMPA subunit in the PFC, amygdala, dorsal hippocampus, and bed nucleus of the stria terminalis [127]. Interestingly, GluR1 phosphorylation increases the trafficking of the subunit to the cell surface, suggesting that cocaine may selectively decrease the excitatory current induced by AMPA in some regions, and OXT is able to correct this abnormality. Thus, while OXT seems to have differential effects on NMDA and AMPA surface expression in certain brain regions, the neuropeptide ultimately opposes drug-induced alterations of ionotropic receptors.

Changes in Glu transmission following OXT administration may also occur because of OXTRs interacting with metabotropic Glu receptors. Specifically, Group 2 mGluRs seem to be highly involved in OXT’s effects. Group 2 inhibitory receptors are involved in regulating the release of Glu and DA in structures involved in reward processing; agonists of these receptors can inhibit reinstatement of drug-seeking behavior [128]. Blocking presynaptic Group 2 receptors in the NAc prevented OXT from reducing cued methamphetamine seeking [129]. Furthermore, an mGluR2/3 antagonist inhibited the effects of intra-accumbal OXT on cued reinstatement of cocaine seeking [30]. Thus, it is possible that OXT stimulates Group 2 mGluRs to reduce the excitatory effects of Glu in the NAc, subsequently reducing activation in other regions like the mPFC and hippocampus and preventing the initiation of drug-seeking behaviors.

As previously discussed, OXTRs are found on glutamatergic neurons within the VTA, suggesting that OXT directly modulates Glu release in VTA neurons [26]. The precise role of these glutamatergic neurons in reward and addiction are not fully known, though studies have found that VTA glutamatergic neurons may modulate DA transmission to the NAc [130] and influence reward-processes via projections to a number of areas including the NAc, PFC, and amygdala [131]. Furthermore, OXTR-expressing projections have shown that they target the LHb [29], a region that receives Glu input from the VTA and is primarily involved in aversive conditioning [132], which may provide another mechanism through which OXT provides a neuromodulatory role in reward processing. Finally, VTA glutamatergic neurons have been shown to promote aversion behavior via projections to GABAergic interneurons within the NAc that subsequently inhibited NAc medium spiny neurons [133]. While it is not yet known which specific VTA subpopulations of glutamatergic neurons express OXTRs, there are a number of possible mechanisms through which OXT could provide a neuromodulatory effect on reward-processes via its effect on Glu transmission within the VTA. 

### 3.2. Glutamate-Dopamine Interactions

It is possible that OXT’s impact on addictive behaviors occurs due to interference with the DA and Glu interactions that occur in the reward circuit during the addiction cycle. This is supported by the fact that OXTRs are on both dopaminergic and glutamatergic neurons in the VTA [29,134]. At specific glutamatergic inputs to VTA dopamine neurons, OXT acts as a filter by selectively inhibiting excitatory synaptic transmission to the VTA through OXTRs alongside endocannabinoid signaling [9]. OXT has a greater gating effect on Glu neurons that fire only occasionally compared to those that fire repeatedly. These mechanisms lead to a decrease in Glu release in VTA DA neurons in the presence of OXT [9]. Glu-mediated DA release is also relevant to processes such as tonic-phasic DA activity; OXT suppression of Glu would allow for less modulation of tonic and phasic firing of DA neurons, which is a key component of reward processing. Similarly, glutamatergic synaptic transmission in the NAc was dampened in the presence of OXT through a presynaptic mechanism involving serotonergic inputs [9]. This modulatory interaction of OXT on Glu transmission in the VTA allows for regulation of both Glu and DA levels through their interactions [135]. 

### 3.3. Glutamate-GABA Interactions

Although there has been no research that looks directly at the interactions between OXT, GABA, and DA levels, it is possible that OXT interacts with GABA neurons that project onto glutamatergic and dopaminergic neurons in the VTA. There are OXTRs found on GABAergic neurons in the VTA [29] and PFC [136]. Additionally, OXT has been shown to decrease alcohol-induced GABAergic signaling in the CeA, and to reduce GABA receptor function in both alcohol-dependent and alcohol-naïve rats [124]. GABAergic neurons innervating the VTA inhibit glutamatergic and dopaminergic neurons and their respective neurotransmitter release [137]. Therefore, a plausible mechanism is that OXT enhances GABA’s inhibitory effects on glutamatergic and dopaminergic VTA neurons, which can then dampen the signaling of these cells and decrease mesocorticolimbic Glu and DA levels. In fact, Qi et al. (2012) demonstrated that OXT increased extracellular GABA levels in the mPFC following methamphetamine administration [123]. Thus, OXT may be acting as an activating neuromodulator in regions where drugs cause an inability to control behavior and impulses.

### 3.4. Changes in Astrocyte Function 

Astrocytes are the most abundant type of glial cell in the brain and have been shown to play an important role in the regulation of Glu and GABA neurotransmission [138]. The activity and physiology of astrocytes can be affected by various drugs of abuse. Cocaine and amphetamines (methamphetamine and 3,4-methylenedioxymethamphetamine (MDMA)) greatly stimulate the activation of astrocytes, which proposes the basis for the neurotoxicity associated with drugs of abuse [139,140]. Drugs such as methamphetamine and morphine cause an increase in the expression of glial fibrillary acidic protein (GFAP), which is a cytoskeleton component in astrocytes that is associated with neurotoxicity and helps to localize astrocytic functional proteins [125,138]. Since astrocytes are essential for the normal functioning of glutamatergic neurons because of their role in Glu reuptake, synthesis, and transmission regulation [141], alterations of astrocytic physiology and function can prove detrimental to glutamatergic signaling in the reward pathway. This dysfunction has implications in addiction specifically in regard to the Glu homeostasis hypothesis and NAc function, because synaptic plasticity in the NAc is regulated through astrocytic-control of extrasynaptic Glu release and reuptake [92]. 

OXT can impact various components of astrocyte physiology. For instance, OXT reduces the drug-induced increase in GFAP expression and alters the neural plasticity associated with the protein [125,126]. A decrease in GFAP also causes a decrease in Glu transport by preventing GLT-1 trafficking to the cell surface [142]. Therefore, these findings suggest an additional explanation for OXT’s mechanism on attenuating drug-associated behaviors: OXT reduces the neurotoxic effects of drugs via a reduction in GFAP levels. Through restoring GFAP expression, and consequently astrocyte function, OXT may indirectly affect glutamatergic transmission in a way that opposes addictive behaviors. By reducing GFAP levels, OXT reduces the trafficking of GLT-1 and consequently lowers the activity of Glu and its excitatory effects. These effects are tied to astrocytes because GLT-1 is exclusively expressed in neural astrocytes [143,144]. An interesting caveat is that upregulation of GLT-1 expression is being researched as a potential therapeutic treatment for addiction due to its ability to facilitate Glu reuptake [145,146].

## 4. Discussion

The exogenous administration of OXT impacts Glu and DA transmission in addiction-associated neural pathways. Due to the significant role of Glu in the addiction cycle, it is likely that the interference with its transmission is how OXT exerts its effects. We suggest that OXT impacts the glutamatergic system by restoring the normal activity of ionotropic and metabotropic Glu receptors, opposing drug-induced changes to Glu/DA and Glu/GABAA interactions, and decreasing GFAP and GLT-1 expression to mend astrocyte function. The evidence provided in this review offers support for these proposed mechanisms, although there does not appear to be just one way that OXT carries out its functions.

With cases of substance-abuse disorders growing each year, there is a dire need for effective pharmacotherapeutics for addiction. However, some have questioned whether the systemic administration of OXT actually allows for the compound to reach the brain in sufficient amounts, due to the challenge posed by the blood–brain barrier (BBB). We maintain that OXT is still highly effective when given peripherally, as demonstrated by numerous studies which have used peripheral injections on rodents to produce an OXT-based effect [10,61]. Further, central administration of an OXT or OXTR antagonist inhibits the effects of peripherally injected OXT [64,147]. Peripherally circulating OXT could be crossing the BBB through carrier-mediated transport or transcellular passage across the endothelial cells that compose the BBB [148]. Another possibility is that the drug reaches the brain through leakages into the cerebral spinal fluid, which occurs when the permeability of the BBB is compromised by addiction, hypertension, stress, or disease [10,148]. Intranasal administration of OXT also seems to effectively deliver the drug to the brain, as this method allows for bypassing the BBB [149]. Being able to avoid the limitations set by the BBB through intranasal administration opens up new opportunities for pharmaceutical treatments of disease. 

Another matter to consider for exploring OXT as a therapeutic for addiction is that studies have not yet shown the localization of OXTRs in the human VTA. There is, however, strong evidence that OXT enhances the activation of the VTA in humans in response to rewarding social stimuli [150,151]. Knowing how central the role of the VTA is in reward processing and addictive behaviors, all of the findings that demonstrate that OXT reduces drug-seeking in humans implies that there must be OXT binding sites on the VTA. It may just be that very few studies have even attempted to localize OXTRs in the human brain [15,152] that this discovery has not been made. Additionally, it is well-established that OXTRs are found in the VTA of rodents [12,16], and given the high similarity between the rodent and human brain, it is not unreasonable to assume that OXTRs exist in the human VTA. The human brain has displayed dense OXTR binding sites in other regions, such as the substantia nigra pars compacta, so this may be a potential target for OXT as well [12]. 

This review described potential mechanisms through which OXT attenuates reward-seeking behaviors and the reinstatement for drugs of abuse and natural rewards. The insights provided in this review add to the growing literature of OXT as a possible therapeutic treatment to reduce addictive behaviors. However, there is still a need for further examination of more specific aspects of OXT’s mechanisms. For instance, it is unknown how significant of a role other compounds such as GABA and endocannabinoids may have in OXT’s mechanisms. Additionally, more studies must be conducted to demonstrate that the mechanistic processes of OXT observed in rodents occur the same way in humans. With a better understanding of how OXT works, its potential uses and long-term effects become clearer. 

It is important to consider the future implications of using OXT to treat addiction. A key factor is the addictive nature of OXT itself, especially to its prosocial and anxiolytic effects. Although studies indicate that it does not induce addictive behaviors unless at exceedingly high levels, if OXT is developed into a drug therapy, there is still the possibility of people consuming the compound at amounts far greater than the appropriate dose. Another factor to consider is the ramifications of OXT administration on the peripheral system, specifically on the reproductive system, where it has a substantial role. Thus, there is still a need to examine certain aspects of OXT being a treatment for addiction. Nonetheless, this is an exciting and novel direction towards pharmacologically disrupting the addiction cycle and the behaviors it induces.

## Figures and Tables

**Table 1:** Oxytocin’s effects on drug-induced molecular changes based on structure. VTA = ventral tegmental area; NAc = nucleus accumbens; mPFC = medial prefrontal cortex; PFC = prefrontal cortex; CeA = central amygdala; SON = supraoptic nuclei; GABA = γ-aminobutyric acid; GFAP = glial fibrillary acidic protein.

Neuroanatomical Structure	Oxytocin’s Effect
mPFC	Decrease extracellular glutamate levels [8]
VTA	Decrease glutamate release [9]
NAc	Decrease dopamine levels [69]
Hippocampus	Increase glutamate levels [123]
Hippocampus, mPFC	Increase extracellular GABA levels [123]
PFC	Decrease NR1 subunit expression [123]
PFC, dorsal hippocampus, amygdala, bed nucleus of stria terminalis	Increase phosphorylation of GluR1 subunit [124]
CeA	Decrease GABA transmission [124]
SON	Decrease GFAP expression [125,126]

## Data Availability

Not applicable.

## References

[1] Leong K.-C., Cox S., King C., Becker H., Reichel C.M. (2018). Oxytocin and Rodent Models of Addiction. Int. Rev. Neurobiol..

[2] McGregor I.S., Bowen M.T. (2012). Breaking the loop: Oxytocin as a potential treatment for drug addiction. Horm. Behav..

[3] Morales-Rivera A., Hernández-Burgos M.M., Martínez-Rivera A., Pérez-Colón J., Rivera R., Montalvo J., Rodríguez-Borrero E., Maldonado-Vlaar C.S. (2014). Anxiolytic effects of oxytocin in cue-induced cocaine seeking behavior in rats. Psychopharmacology.

[4] Lee M.R., Rohn M.C.H., Tanda G., Leggio L. (2016). Targeting the Oxytocin System to Treat Addictive Disorders: Rationale and Progress to Date. CNS Drugs.

[5] Bethlehem R.A.I., Baron-Cohen S., van Honk J., Auyeung B., Bos P.A. (2014). The oxytocin paradox. Front. Behav. Neurosci..

[6] Love T.M. (2014). Oxytocin, motivation and the role of dopamine. Pharmacol. Biochem. Behav..

[7] Sarnyai Z., Kovacs G.H. (2014). Oxytocin in learning and addiction: From early discoveries to the present. Pharmacol. Biochem. Behav..

[8] Qi J., Yang J.-Y., Wang F., Zhao Y.-N., Song M., Wu C.-F. (2009). Effects of oxytocin on methamphetamine-induced conditioned place preference and the possible role of glutamatergic neurotransmission in the medial prefrontal cortex of mice in reinstatement. Neuropharmacology.

[9] Xiao L., Priest M.F., Kozorovitskiy Y. (2018). Oxytocin functions as a spatiotemporal filter for excitatory synaptic inputs to VTA dopamine neurons. Elife.

[10] Buisman-Pijlman F.T.A., Sumracki N.M., Gordon J.J., Hull P.R., Carter C.S., Tops M. (2014). Individual differences underlying susceptibility to addiction: Role for the endogenous oxytocin system. Pharmacol. Biochem. Behav..

[11] Kiss A., Mikkelsen J.D. (2005). Oxytocin--anatomy and functional assignments: A minireview. Endocr. Regul..

[12] Gimpl G., Fahrenholz F. (2001). The oxytocin receptor system: Structure, function, and regulation. Physiol. Rev..

[13] Alves E., Fielder A., Ghabriel N., Sawyer M., Buisman-Pijlman F.T.A. (2015). Early social environment affects the endogenous oxytocin system: A review and future directions. Front. Endocrinol..

[14] Holubová A., Poništ S., Jurčovičová J., Šlamberová R. (2019). Different Oxytocin Responses to Acute Methamphetamine Treatment in Juvenile Female Rats Perinatally Exposed to Stress and/or Methamphetamine Administration. Front. Physiol..

[15] Boccia M.L., Petrusz P., Suzuki K., Marson L., Pedersen C.A. (2013). Immunohistochemical localization of oxytocin receptors in human brain. Neuroscience.

[16] Vaccari C., Lolait S.J., Ostrowski N.L. (1998). Comparative distribution of vasopressin V1b and oxytocin receptor messenger ribonucleic acids in brain. Endocrinology.

[17] Ninan I. (2011). Oxytocin suppresses basal glutamatergic transmission but facilitates activity-dependent synaptic potentiation in the medial prefrontal cortex. J. Neurochem..

[18] Dumais K.M., Veenema A.H. (2016). Vasopressin and oxytocin receptor systems in the brain: Sex differences and sex-specific regulation of social behavior. Front. Neuroendocrinol..

[19] Adinoff B. (2004). Neurobiologic processes in drug reward and addiction. Harv. Rev. Psychiatry.

[20] Russo S.J., Nestler E.J. (2013). The brain reward circuitry in mood disorders. Nat. Rev. Neurosci..

[21] Cooper S., Robison A.J., Mazei-Robison M.S. (2017). Reward Circuitry in Addiction. Neurotherapeutics.

[22] Hnasko T.S., Hjelmstad G.O., Fields H.L., Edwards R.H. (2012). Ventral tegmental area glutamate neurons: Electrophysiological properties and projections. J. Neurosci..

[23] Volkow N.D., Fowler J.S., Wang G.J. (2004). The addicted human brain viewed in the light of imaging studies: Brain circuits and treatment strategies. Neuropharmacology.

[24] Galvan A., Hare T.A., Davidson M., Spicer J., Glover G., Casey B.J. (2005). The role of ventral frontostriatal circuitry in reward-based learning in humans. J. Neurosci..

[25] Nusslock R., Alloy L.B. (2017). Reward processing and mood-related symptoms: An RDoC and translational neuroscience perspective. J. Affect. Disord..

[26] Haber S.N., Knutson B. (2010). The reward circuit: Linking primate anatomy and human imaging. Neuropsychopharmacology.

[27] Sofroniew M.V. (1980). Projections from vasopressin, oxytocin, and neurophysin neurons to neural targets in the rat and human. J. Histochem. Cytochem..

[28] Xiao L., Priest M.F., Nasenbeny J., Lu T., Kozorovitskiy Y. (2017). Biased Oxytocinergic Modulation of Midbrain Dopamine Systems. Neuron.

[29] Peris J., MacFadyen K., Smith J.A., de Kloet A.D., Wang L., Krause E.G. (2017). Oxytocin receptors are expressed on dopamine and glutamate neurons in the mouse ventral tegmental area that project to nucleus accumbens and other mesolimbic targets. J. Comp. Neurol..

[30] Weber R.A., Logan C.N., Leong K.-C., Peris J., Knackstedt L., Reichel C.M. (2018). Regionally Specific Effects of Oxytocin on Reinstatement of Cocaine Seeking in Male and Female Rats. Int. J. Neuropsychopharmacol..

[31] Goodman A. (2008). Neurobiology of addiction. An integrative review. Biochem. Pharmacol..

[32] Olsen C.M. (2011). Natural rewards, neuroplasticity, and non-drug addictions. Neuropharmacology.

[33] Kelley A.E., Berridge K.C. (2002). The neuroscience of natural rewards: Relevance to addictive drugs. J. Neurosci..

[34] Flagel S.B., Akil H., Robinson T.E. (2009). Individual differences in the attribution of incentive salience to reward-related cues: Implications for addiction. Neuropharmacology.

[35] Simmons J.M., Richmond B.J. (2008). Dynamic changes in representations of preceding and upcoming reward in monkey orbitofrontal cortex. Cereb. Cortex.

[36] Laricchiuta D., Petrosini L. (2014). Individual differences in response to positive and negative stimuli: Endocannabinoid-based insight on approach and avoidance behaviors. Front. Syst. Neurosci..

[37] McClure S.M., York M.K., Montague P.R. (2004). The neural substrates of reward processing in humans: The modern role of FMRI. Neuroscientist.

[38] Carter R.M., Macinnes J.J., Huettel S.A., Adcock R.A. (2009). Activation in the VTA and nucleus accumbens increases in anticipation of both gains and losses. Front. Behav. Neurosci..

[39] Zellner M.R., Ranaldi R. (2010). How conditioned stimuli acquire the ability to activate VTA dopamine cells: A proposed neurobiological component of reward-related learning. Neurosci. Biobehav. Rev..

[40] van Zessen R., Phillips J.L., Budygin E.A., Stuber G.D. (2012). Activation of VTA GABA neurons disrupts reward consumption. Neuron.

[41] Kosten T.R., George T.P. (2002). The neurobiology of opioid dependence: Implications for treatment. Sci. Pract. Perspect..

[42] Kovacs G.L., Telegdy G. (1987). Beta-endorphin tolerance is inhibited by oxytocin. Pharmacol. Biochem. Behav..

[43] Corbit L.H., Muir J.L., Balleine B.W. (2001). The role of the nucleus accumbens in instrumental conditioning: Evidence of a functional dissociation between accumbens core and shell. J. Neurosci..

[44] Scofield M.D., Heinsbroek J.A., Gipson C.D., Kupchik Y.M., Spencer S., Smith A.C.W., Roberts-Wolfe D., Kalivas P.W. (2016). The Nucleus Accumbens: Mechanisms of Addiction across Drug Classes Reflect the Importance of Glutamate Homeostasis. Pharmacol. Rev..

[45] Sesack S.R., Grace A.A. (2010). Cortico-Basal Ganglia reward network: Microcircuitry. Neuropsychopharmacology.

[46] Shiflett M.W. (2011). Balleine, Molecular substrates of action control in cortico-striatal circuits. Prog. Neurobiol..

[47] De Luca M.A. (2014). Habituation of the responsiveness of mesolimbic and mesocortical dopamine transmission to taste stimuli. Front. Integr. Neurosci..

[48] Bimpisidis Z., De Luca M.A., Pisanu A., Di Chiara G. (2013). Lesion of medial prefrontal dopamine terminals abolishes habituation of accumbens shell dopamine responsiveness to taste stimuli. Eur. J. Neurosci..

[49] Di Chiara G., Bassareo V., Fenu S., De Luca M.A., Spina L., Cadoni C., Acquas E., Carboni E., Valentini V., Lecca D. (2004). Dopamine and drug addiction: The nucleus accumbens shell connection. Neuropharmacology.

[50] Ito R., Robbins T.W., Everitt B.J. (2004). Differential control over cocaine-seeking behavior by nucleus accumbens core and shell. Nat. Neurosci..

[51] Koob G.F., Caine B., Markou A., Pulvirenti L., Weiss F. (1994). Role for the mesocortical dopamine system in the motivating effects of cocaine. NIDA Res. Monogr..

[52] Ritz M.C., Lamb R.J., Goldberg S.R., Kuhar M.J. (1987). Cocaine receptors on dopamine transporters are related to self-administration of cocaine. Science.

[53] Moszczynska A. (2016). Neurobiology and Clinical Manifestations of Methamphetamine Neurotoxicity. Psychiatr. Times.

[54] Chiu V.M., Schenk J.O. (2012). Mechanism of action of methamphetamine within the catecholamine and serotonin areas of the central nervous system. Curr. Drug Abuse Rev..

[55] Abrahao K.P., Salinas A.G., Lovinger D.M. (2017). Alcohol and the Brain: Neuronal Molecular Targets, Synapses, and Circuits. Neuron.

[56] Volkow N.D., Wang G.-J., Maynard L., Fowler J.S., Jayne B., Telang F., Logan J., Ding Y.-S., Gatley S.J., Hitzemann R. (2002). Effects of alcohol detoxification on dopamine D2 receptors in alcoholics: A preliminary study. Psychiatry Res..

[57] Zhu P.J., Lovinger D.M. (2006). Ethanol potentiates GABAergic synaptic transmission in a postsynaptic neuron/synaptic bouton preparation from basolateral amygdala. J. Neurophysiol..

[58] De Luca M.A., Buczynski M.W., di Chiara G. (2018). Loren Parsons’ contribution to addiction neurobiology. Addict. Biol..

[59] Sarnyai Z. (1998). Oxytocin and neuroadaptation to cocaine. Prog Brain Res..

[60] Zhou L., Ghee S.M., See R.E., Reichel C.M. (2015). Oxytocin differentially affects sucrose taking and seeking in male and female rats. Behav. Brain Res..

[61] Carson D.S., Cornish J.L., Guastella A.J., Hunt G.E., McGregor I.S. (2010). Oxytocin decreases methamphetamine self-administration, methamphetamine hyperactivity, and relapse to methamphetamine-seeking behaviour in rats. Neuropharmacology.

[62] Baracz S.J., Rourke P.I., Pardey M.C., Hunt G.E., McGregor I.S., Cornish J.L. (2012). Oxytocin directly administered into the nucleus accumbens core or subthalamic nucleus attenuates methamphetamine-induced conditioned place preference. Behav. Brain Res..

[63] Cox B.M., Young A.B., See R.E., Reichel C.M. (2013). Sex differences in methamphetamine seeking in rats: Impact of oxytocin. Psychoneuroendocrinology.

[64] Cox B.M., Bentzley B.S., Regen-Tuero H., See R.E., Reichel C.M., Aston-Jones G. (2017). Oxytocin Acts in Nucleus Accumbens to Attenuate Methamphetamine Seeking and Demand. Biol. Psychiatry.

[65] Baracz S.J., Everett N.A., McGregor I.S., Cornish J.L. (2016). Oxytocin in the nucleus accumbens core reduces reinstatement of methamphetamine-seeking behaviour in rats. Addict. Biol..

[66] Zanos P., Georgiou P., Weber C., Robinson F., Kouimtsidis C., Niforooshan R., Bailey A. (2018). Oxytocin and opioid addiction revisited: Old drug, new applications. Br. J. Pharmacol..

[67] Kovacs G.L., Borthaiser Z., Telegdy G. (1985). Oxytocin reduces intravenous heroin self-administration in heroin-tolerant rats. Life Sci..

[68] Kovacs G.L., Sarnyai Z., Szabo G. (1998). Oxytocin and addiction: A review. Psychoneuroendocrinology.

[69] Peters S.T., Bowen M.T., Bohrer K., McGregor I.S., Neumann I.D. (2017). Oxytocin inhibits ethanol consumption and ethanol-induced dopamine release in the nucleus accumbens. Addict. Biol..

[70] Arletti R., Benelli A., Bertolini A. (1989). Influence of oxytocin on feeding behavior in the rat. Peptides.

[71] Mullis K., Kay K., Williams D.L. (2013). Oxytocin action in the ventral tegmental area affects sucrose intake. Brain Res..

[72] Patel D., Sundar M., Lorenz E. (2020). Oxytocin Attenuates Expression, but Not Acquisition, of Sucrose Conditioned Place Preference in Rats. Front. Behav. Neurosci..

[73] Pedersen C.A., Smedley K.L., Leserman J., Jarskog L.F., Rau S.W., Kampov-Polevoi A., Casey R.L., Fender T., Garbutt J.C. (2013). Intranasal oxytocin blocks alcohol withdrawal in human subjects. Alcohol. Clin. Exp. Res..

[74] McRae-Clark A.L., Baker N.L., Maria M.M.-S., Brady K.T. (2013). Effect of oxytocin on craving and stress response in marijuana-dependent individuals: A pilot study. Psychopharmacology.

[75] D’Souza M.S. (2015). Glutamatergic transmission in drug reward: Implications for drug addiction. Front. Neurosci..

[76] Niciu M.J., Kelmendi B., Sanacora G. (2012). Overview of glutamatergic neurotransmission in the nervous system. Pharmacol. Biochem. Behav..

[77] Conn P.J., Pin J.P. (1997). Pharmacology and functions of metabotropic glutamate receptors. Annu. Rev. Pharmacol. Toxicol..

[78] Crupi R., Impellizzeri D., Cuzzocrea S. (2019). Role of Metabotropic Glutamate Receptors in Neurological Disorders. Front. Mol. Neurosci..

[79] Cartmell J., Schoepp D.D. (2000). Regulation of neurotransmitter release by metabotropic glutamate receptors. J. Neurochem..

[80] Kenny P.J., Markou A. (2004). The ups and downs of addiction: Role of metabotropic glutamate receptors. Trends Pharmacol. Sci..

[81] Schoepp D.D. (2001). Unveiling the functions of presynaptic metabotropic glutamate receptors in the central nervous system. J. Pharmacol. Exp. Ther..

[82] McFarland K., Kalivas P.W. (2001). The circuitry mediating cocaine-induced reinstatement of drug-seeking behavior. J. Neurosci..

[83] Brog J.S., Salyapongse A., Deutch A.Y., Zahm D.S. (1993). The patterns of afferent innervation of the core and shell in the “accumbens” part of the rat ventral striatum: Immunohistochemical detection of retrogradely transported fluoro-gold. J. Comp. Neurol..

[84] Kalivas P.W., Volkow N., Seamans J. (2005). Unmanageable motivation in addiction: A pathology in prefrontal-accumbens glutamate transmission. Neuron.

[85] McFarland K., Lapish C.C., Kalivas P.W. (2003). Prefrontal glutamate release into the core of the nucleus accumbens mediates cocaine-induced reinstatement of drug-seeking behavior. J. Neurosci..

[86] Tzschentke T.M., Schmidt W.J. (2003). Glutamatergic mechanisms in addiction. Mol. Psychiatry.

[87] Di Ciano P., Everitt B.J. (2001). Dissociable effects of antagonism of NMDA and AMPA/KA receptors in the nucleus accumbens core and shell on cocaine-seeking behavior. Neuropsychopharmacology.

[88] Cornish J.L., Kalivas P.W. (2000). Glutamate transmission in the nucleus accumbens mediates relapse in cocaine addiction. J. Neurosci..

[89] Fuchs R.A., Evans K.A., Parker M.C., See R.E. (2004). Differential involvement of the core and shell subregions of the nucleus accumbens in conditioned cue-induced reinstatement of cocaine seeking in rats. Psychopharmacology.

[90] Gibson G.D., Millan E.Z., McNally G.P. (2019). The nucleus accumbens shell in reinstatement and extinction of drug seeking. Eur. J. Neurosci..

[91] Park W.-K., Bari A.A., Jey A.R., Anderson S.M., Spealman R.D., Rowlett J.K., Pierce R.C. (2002). Cocaine administered into the medial prefrontal cortex reinstates cocaine-seeking behavior by increasing AMPA receptor-mediated glutamate transmission in the nucleus accumbens. J. Neurosci..

[92] Kalivas P.W. (2009). The glutamate homeostasis hypothesis of addiction. Nat. Rev. Neurosci..

[93] Reissner K.J., Kalivas P.W. (2010). Using glutamate homeostasis as a target for treating addictive disorders. Behav. Pharmacol..

[94] Baker D.A., McFarland K., Lake R.W., Shen H., Tang X.-C., Toda S., Kalivas P.W. (2003). Neuroadaptations in cystine-glutamate exchange underlie cocaine relapse. Nat. Neurosci..

[95] Juarez B., Han M.H. (2016). Diversity of Dopaminergic Neural Circuits in Response to Drug Exposure. Neuropsychopharmacology.

[96] Sibley D.R., Monsma F.J. (1992). Molecular biology of dopamine receptors. Trends Pharmacol. Sci..

[97] Ford C.P. (2014). The role of D2-autoreceptors in regulating dopamine neuron activity and transmission. Neuroscience.

[98] Geisler S., Wise R.A. (2008). Functional implications of glutamatergic projections to the ventral tegmental area. Rev. Neurosci..

[99] Geisler S., Zahm D.S. (2005). Afferents of the ventral tegmental area in the rat-anatomical substratum for integrative functions. J. Comp. Neurol..

[100] Watabe-Uchida M., Zhu L., Ogawa S.K., Vamanrao A., Uchida N. (2012). Whole-brain mapping of direct inputs to midbrain dopamine neurons. Neuron.

[101] Overton P.G., Clark D. (1997). Burst firing in midbrain dopaminergic neurons. Brain Res. Brain Res. Rev..

[102] Stamatakis A.M., Jennings J.H., Ung R.L., Blair G.A., Weinberg R.J., Neve R.L., Boyce F., Mattis J., Ramakrishnan C., Deisseroth K. (2013). A unique population of ventral tegmental area neurons inhibits the lateral habenula to promote reward. Neuron.

[103] Floresco S.B., Todd C.L., Grace A.A. (2001). Glutamatergic afferents from the hippocampus to the nucleus accumbens regulate activity of ventral tegmental area dopamine neurons. J. Neurosci..

[104] Hu G., Duffy P., Swanson C., Ghasemzadeh M.B., Kalivas P.W. (1999). The regulation of dopamine transmission by metabotropic glutamate receptors. J. Pharmacol. Exp. Ther..

[105] Taber M.T., Fibiger H.C. (1995). Electrical stimulation of the prefrontal cortex increases dopamine release in the nucleus accumbens of the rat: Modulation by metabotropic glutamate receptors. J. Neurosci..

[106] Borland L.M., Michael A.C. (2004). Voltammetric study of the control of striatal dopamine release by glutamate. J. Neurochem..

[107] Mount H., Quirion R., Chaudieu I., Boksa P. (1990). Stimulation of dopamine release from cultured rat mesencephalic cells by naturally occurring excitatory amino acids: Involvement of both N-methyl-D-aspartate (NMDA) and non-NMDA receptor subtypes. J. Neurochem..

[108] Pierce R.C., Born B., Adams M., Kalivas P.W. (1996). Repeated intra-ventral tegmental area administration of SKF-38393,induces behavioral and neurochemical sensitization to a subsequent cocaine challenge. J. Pharmacol. Exp. Ther..

[109] Chao S.Z., Ariano M.A., Peterson D.A., Wolf M.E. (2002). D1 dopamine receptor stimulation increases GluR1 surface expression in nucleus accumbens neurons. J. Neurochem..

[110] Ladepeche L., Yang L., Bouchet D., Groc L. (2013). Regulation of dopamine D1 receptor dynamics within the postsynaptic density of hippocampal glutamate synapses. PLoS ONE.

[111] Martin G., Nie Z., Siggins G.R. (1997). mu-Opioid receptors modulate NMDA receptor-mediated responses in nucleus accumbens neurons. J. Neurosci..

[112] Mishra D., Pena-Bravo J.I., Leong K.-C., Lavin A., Reichel C.M. (2017). Methamphetamine self-administration modulates glutamate neurophysiology. Brain Struct. Funct..

[113] Mulholland P.J., Chandler L.J., Kalivas P.W. (2016). Signals from the Fourth Dimension Regulate Drug Relapse. Trends Neurosci..

[114] Spencer S., Scofield M., Kalivas P.W. (2016). The good and bad news about glutamate in drug addiction. J. Psychopharmacol..

[115] Wolf M.E., Ferrario C.R. (2010). AMPA receptor plasticity in the nucleus accumbens after repeated exposure to cocaine. Neurosci. Biobehav. Rev..

[116] Luscher C., Malenka R.C. (2011). Drug-evoked synaptic plasticity in addiction: From molecular changes to circuit remodeling. Neuron.

[117] Vanderschuren L.J., Kalivas P.W. (2000). Alterations in dopaminergic and glutamatergic transmission in the induction and expression of behavioral sensitization: A critical review of preclinical studies. Psychopharmacology.

[118] Wolf M.E. (1998). The role of excitatory amino acids in behavioral sensitization to psychomotor stimulants. Prog. Neurobiol..

[119] Dong Y., Nestler E.J. (2014). The neural rejuvenation hypothesis of cocaine addiction. Trends Pharmacol. Sci..

[120] Kalivas P.W., Volkow N.D. (2005). The neural basis of addiction: A pathology of motivation and choice. Am. J. Psychiatry.

[121] Tang Y., Chen Z., Tao H., Li C., Zhang X., Tang A., Liu Y. (2014). Oxytocin activation of neurons in ventral tegmental area and interfascicular nucleus of mouse midbrain. Neuropharmacology.

[122] Peris J., Steck M.R., Krause E.G. (2020). Oxytocin treatment for alcoholism: Potential neurocircuitry targets. Neuropharmacology.

[123] Qi J., Han W.-Y., Yang J.-Y., Wang L.-H., Dong Y.-X., Wang F., Song M., Wu C.-F. (2012). Oxytocin regulates changes of extracellular glutamate and GABA levels induced by methamphetamine in the mouse brain. Addict. Biol..

[124] Tunstall B.J., Kirson D., Zallar L.J., McConnell S.A., Vendruscolo J.C.M., Ho C.P., Oleata C.S., Khom S., Manning M., Lee M.R. (2019). Oxytocin blocks enhanced motivation for alcohol in alcohol dependence and blocks alcohol effects on GABAergic transmission in the central amygdala. PLoS Biol..

[125] Wang P., Qin D., Wang Y.F. (2017). Oxytocin Rapidly Changes Astrocytic GFAP Plasticity by Differentially Modulating the Expressions of pERK 1/2 and Protein Kinase A. Front. Mol. Neurosci..

[126] Wang Y.F., Hatton G.I. (2009). Astrocytic plasticity and patterned oxytocin neuronal activity: Dynamic interactions. J. Neurosci..

[127] Zhou L., Sun W.-L., Young A.B., Lee K., McGinty J.F., See R.E. (2014). Oxytocin reduces cocaine seeking and reverses chronic cocaine-induced changes in glutamate receptor function. Int. J. Neuropsychopharmacol..

[128] Moussawi K., Kalivas P.W. (2010). Group II metabotropic glutamate receptors (mGlu2/3) in drug addiction. Eur. J. Pharmacol..

[129] Bernheim A., Leong K.-C., Berini C., Reichel C.M. (2017). Antagonism of mGlu2/3 receptors in the nucleus accumbens prevents oxytocin from reducing cued methamphetamine seeking in male and female rats. Pharmacol. Biochem. Behav..

[130] Britt J.P., Benaliouad F., McDevitt R.A., Stuber G.D., Wise R.A., Bonci A. (2012). Synaptic and behavioral profile of multiple glutamatergic inputs to the nucleus accumbens. Neuron.

[131] Taylor S.R., Badurek S., Dileone R.J., Nashmi R., Minichiello L., Picciotto M.R. (2014). GABAergic and glutamatergic efferents of the mouse ventral tegmental area. J. Comp. Neurol..

[132] Root D.H., Mejias-Aponte C.A., Qi J., Morales M. (2014). Role of glutamatergic projections from ventral tegmental area to lateral habenula in aversive conditioning. J. Neurosci..

[133] Qi J., Zhang S., Wang H.L., Barker D.J., Miranda-Barrientos J., Morales M. (2016). VTA glutamatergic inputs to nucleus accumbens drive aversion by acting on GABAergic interneurons. Nat. Neurosci..

[134] Baskerville T.A., Allard J., Wayman C., Douglas A.J. (2009). Dopamine-oxytocin interactions in penile erection. Eur. J. Neurosci..

[135] Chen M., Zhao Y., Yang H., Luan W., Song J., Cui D., Dong Y., Lai B., Ma L., Zheng P. (2015). Morphine disinhibits glutamatergic input to VTA dopamine neurons and promotes dopamine neuron excitation. Elife.

[136] Tan Y., Singhal S.M., Harden S.W., Cahill K.M., Nguyen D.T.M., Colon-Perez L.M., Sahagian T.J., Thinschmidt J.S., de Kloet A.D., Febo M. (2019). Oxytocin Receptors Are Expressed by Glutamatergic Prefrontal Cortical Neurons That Selectively Modulate Social Recognition. J. Neurosci..

[137] Yu X., Li W., Ma Y., Tossell K., Harris J.J., Harding E.C., Ba W., Miracca G., Wang D., Li L. (2019). GABA and glutamate neurons in the VTA regulate sleep and wakefulness. Nat. Neurosci..

[138] Miguel-Hidalgo J.J. (2009). The role of glial cells in drug abuse. Curr. Drug Abuse Rev..

[139] Fattore L., Puddu M.C., Picciau S., Cappai A., Fratta W., Serra G.P., Spiga S. (2002). Astroglial in vivo response to cocaine in mouse dentate gyrus: A quantitative and qualitative analysis by confocal microscopy. Neuroscience.

[140] Itzhak Y., Achat-Mendes C. (2004). Methamphetamine and MDMA (ecstasy) neurotoxicity: ‘of mice and men’. IUBMB Life.

[141] Hertz L., Zielke H.R. (2004). Astrocytic control of glutamatergic activity: Astrocytes as stars of the show. Trends Neurosci..

[142] Hughes E.G., Maguire J.L., McMinn M.T., Scholz R.E., Sutherland M.L. (2004). Loss of glial fibrillary acidic protein results in decreased glutamate transport and inhibition of PKA-induced EAAT2 cell surface trafficking. Brain Res. Mol. Brain Res..

[143] Lehre K.P., Levy L.M., Ottersen O.P., Storm-Mathisen J., Danbolt N.C. (1995). Differential expression of two glial glutamate transporters in the rat brain: Quantitative and immunocytochemical observations. J. Neurosci..

[144] Rothstein J.D., Martin L., Levey A.I., Dykes-Hoberg M., Jin L., Wu D., Nash N., Kuncl R.W. (1994). Localization of neuronal and glial glutamate transporters. Neuron.

[145] Rao P., Yallapu M.M., Sari Y., Fisher P.B., Kumar S. (2015). Designing Novel Nanoformulations Targeting Glutamate Transporter Excitatory Amino Acid Transporter 2: Implications in Treating Drug Addiction. J. Pers. Nanomed..

[146] Takahashi K., Foster J.B., Lin C.L. (2015). Glutamate transporter EAAT2: Regulation, function, and potential as a therapeutic target for neurological and psychiatric disease. Cell Mol. Life Sci..

[147] Sarnyai Z., Babarczy E., Krivan M., Szabo G., Kovacs G.L., Barth T., Telegdy G. (1991). Selective attenuation of cocaine-induced stereotyped behaviour by oxytocin: Putative role of basal forebrain target sites. Neuropeptides.

[148] McEwen B.B. (2004). Brain-fluid barriers: Relevance for theoretical controversies regarding vasopressin and oxytocin memory research. Adv. Pharmacol..

[149] Veening J.G., Olivier B. (2013). Intranasal administration of oxytocin: Behavioral and clinical effects, a review. Neurosci. Biobehav. Rev..

[150] Groppe S.E., Gossen A., Rademacher L., Hahn A., Westphal L., Gründer G., Spreckelmeyer K.N. (2013). Oxytocin influences processing of socially relevant cues in the ventral tegmental area of the human brain. Biol. Psychiatry.

[151] Scheele D., Wille A., Kendrick K.M., Stoffel-Wagner B., Becker B., Güntürkün O., Maier W., Hurlemann R. (2013). Oxytocin enhances brain reward system responses in men viewing the face of their female partner. Proc. Natl. Acad. Sci. USA.

[152] Loup F., Tribollet E., Dubois-Dauphin M., Dreifuss J.J. (1991). Localization of high-affinity binding sites for oxytocin and vasopressin in the human brain. An autoradiographic study. Brain Res..

